# Secoisolariciresinol Diglucoside Exerts Anti-Inflammatory and Antiapoptotic Effects through Inhibiting the Akt/I*κ*B/NF-*κ*B Pathway on Human Umbilical Vein Endothelial Cells

**DOI:** 10.1155/2020/3621261

**Published:** 2020-06-24

**Authors:** Shaoyang Zhang, Meili Cheng, Zhen Wang, Yuzhi Liu, Yuhua Ren, Shikuo Rong, Xue Wang

**Affiliations:** ^1^Department of Cardiology, Liaocheng People's Hospital, Liaocheng, Shandong, China; ^2^College of Clinical Medicine, Ningxia Medical University, Yinchuan, Ningxia, China; ^3^Department of General Surgery, Chengdu Second People's Hospital, Chengdu, Sichuan, China

## Abstract

Inflammation is a key regulator in the progression of atherosclerosis (AS) which extremely affects people's health. Secoisolariciresinol diglucoside (SDG), a plant lignan, is relevant to angiogenesis and cardioprotection against ischemia-reperfusion injury and improves vascular disorders. However, the effect of SDG on cardiovascular disorder is not clear. In the present study, we aimed to investigate the effects of SDG on lipopolysaccharide- (LPS-) stimulated Human Umbilical Vein Endothelial Cells (HUVECs) and elucidate the underlying mechanism. The LPS-stimulated HUVEC cellular model was established. The cell viability, the cell tube formation activity, the nitric oxide (NO) release, the levels of inflammatory cytokine interleukin-1*β* (IL-1*β*), interleukin-6 (IL-6), tumor necrosis factor-*α* (TNF-*α*), the activation of nuclear factor kappa-B (NF-*κ*B) pathway, and the expression of protein kinase B (Akt) were determined using Cell Counting Kit-8, cell tube-formation assay, western blotting, and enzyme-linked immunosorbent assay. Our results revealed that SDG reduces the angiogenic capacity of HUVECs and inhibited LPS-mediated HUVEC injury and apoptosis. In addition, SDG increased NO release and decreased the levels of IL-1*β*, IL-6, and TNF-*α* in LPS-treated HUVECs. Meanwhile, SDG inhibited the NF-*κ*B pathway and downregulated Akt expression in LPS-induced HUVECs. Our results indicated that SDG relieves LPS-mediated HUVEC injury by inhibiting the NF-*κ*B pathway which is partly dependent on the disruption of Akt activation. Therefore, SDG exerts its cytoprotective effects in the context of LPS-treated HUVECs via regulation of the Akt/I*κ*B/NF-*κ*B pathway and may be a potential treatment drug for cardiovascular disease.

## 1. Introduction

Atherosclerosis (AS) is the underlying cause of cardiovascular and cerebrovascular disease, resulting in high rates of mortality worldwide [1]. Inflammation can alter arterial biology of atherosclerosis and produce a systemic environment conducive to AS and finally leads to AS and aggravate the disease [2]. Meanwhile, inflammation can drive arterial hyperplasia, regulate plaque formation, and trigger thrombotic complications of atherosclerosis [3]. The damage caused by inflammation and its associated cell apoptosis in endothelial cells provides important insights into the initiation of AS lesions [4]. Developing new drugs to treat AS through the inflammatory route is a good direction.

Dysregulation of the immune inflammatory response system in vascular endothelial cells produces proinflammatory cytokines and immune protein mediators that trigger AS [5]. Repeated stimulation of diseases such as hyperlipidemia and hypertension may cause aseptic inflammation of vascular endothelial cells, thereby releasing the proinflammatory cytokines IL-1*β*, IL-6, TNF-*α*, and other adhesion molecules and chemokines [6]. In addition, it also causes oxidative stress to promote NO release. The proinflammatory factors, protein mediators, adhesion molecules, and other factors will aggravate the damage of endothelial cells and then trigger or aggravate the process of AS [7]. NF-*κ*B, associated with innate and adaptive immunity as well as in inflammatory diseases, regulates the proinflammatory cytokines and NO release [8]. NF-*κ*B is transferred to the nucleus for transcription to regulate the immune response which is dependent on the phosphorylation of I*κ*B [9]. Studies have confirmed that NF-*κ*B regulates the development of AS disease [10]. Therefore, the I*κ*B/NF-*κ*B pathway is closely associated with the occurrence and development of AS and AS-related diseases.

The secoisolariciresinol diglucoside (SDG), a plant lignan isolated from flaxseed, exerts a cytoprotective effect. Studies have confirmed that SDG suppresses dextran sulfate sodium salt-induced colitis [11], serves as an anti-inflammatory and barrier-protective agent in neuroinflammation [12], and exerts antihypercholesterolemic [13] and also angiogenesis and cardioprotection against ischemia-reperfusion injury [14]. In addition, SDG are effective in lowering blood pressure in individuals with hypertension and metabolic syndrome [15] and exert prevention and treatment of arrhythmias and ventricular remodeling postmyocardial infarction [16]. All these studies confirm the role of SDG in inflammation and cardiovascular disease. Therefore, it can be used as a potential substitute for the treatment of cardiovascular disease. However, the protective effect of SDG and its underlying mechanisms on LPS-induced cell injury in HUVECs remain unknown. In the present study, we established a LPS-stimulated HUVEC cellular injury model to investigate the effects and mechanism of SDG on AS. The cell viability, injury, apoptosis, and the level of inflammatory cytokines were detected. To determine SDG's related mechanism of action, we evaluated the effect of SDG on the Akt/I*κ*B/NF-*κ*B pathway in LPS-induced HUVEC cells.

## 2. Materials and Methods

### 2.1. Reagents

SDG and HY-10249A were purchased from MedChemExpress (New Jersey, USA). Matrigel (354230) and Endothelial Cell Growth Supplement (ECGS) were purchased from Becton, Dickinson and Company (Franklin Lake, New Jersey, USA). Primary antibodies of NF-*κ*B p65 (ab16502) were purchased from Abcam (San Francisco, CA, USA). Primary antibodies of p-NF-*κ*B p65 (3033S), p-I*κ*B-*α* (2859S), and I*κ*B-*α* (4812S) were purchased from Cell Signaling Technology (Boston, USA). Primary antibodies of p-Akt (bs-0876R) and *β*-actin (bs-0061R) were purchased from Bioss Biotechnology Co., Ltd. (Woburn, MA). Other general agents were commercially available.

### 2.2. Cell Lines and Culture Conditions

The Human Umbilical Vein Endothelial Cells (HUVECs) (ATCC, Manassas, VA, USA) cultured in ECM basal medium (Carlsbad, CA, USA) contain 10% fetal bovine serum (FBS) (Gibco, MD, USA) and 1% ECGS at 37°C in 5% CO_2_.

Cells were divided into four groups: the LPS group received LPS; the LPS+SDG group received SDG, followed by LPS. The SDG group received SDG alone; the control group received Dimethyl Sulfoxide (DMSO, 1%) followed by PBS.

### 2.3. Cell Viability

The HUVECs were seeded into 96-well plates at a density of 4 × 10^3^ per well with SDG at different concentrations (lower doses: 0, 100, 250, 500, 750, and 1000 nM; higher doses: 1, 5, 10, 20, 40, 80, and 160 *μ*M) in the absence of FBS. After 24 h, cell viability was determined by CCK-8 assay. In brief, 100 *μ*L medium that contains 10% CCK was added to incubate the cells for 2 h at 37°C. Then, the optical density was detected using a microplate reader at 580 nm.

### 2.4. Endothelial Cell Tube-Formation Assay

HUVECs (2 × 10^4^ cells/well) were cultured on Matrigel matrix (50 *μ*L) preadded to 96-well plates at 37°C. After 6 h, the endothelial cell tube-formation activity was observed. Images were obtained by a microscope (Leica, Oskar, Germany). Tube length was measured using Image J software (Bethesda, MD, USA), and tube formation was expressed as a percentage of the control group.

### 2.5. NO Content Determination

The HUVECs were treated with SDG or DMSO (1%) for 1 h and then treated with LPS or PBS for 24 h. The supernatants were collected to assay for NO levels according to the manufacturer's protocol. The absorbance was measured at 540 nm in a microplate reader.

### 2.6. Western Blotting Analysis

The HUVECs were treated with SDG or DMSO (1%) for 1 h and then treated with LPS or PBS for 6 h. Total or nuclear proteins of colonic tissue or cells were prepared and extracted using the Protein Extraction Kit (KeyGEN Biotechnology Co., Ltd., Jiangsu, China). Then, the concentration of protein was measured using the BCA Protein Assay Kit (KGP902). 50 *μ*g protein was resolved by a 10% sodium dodecyl sulfate- (SDS-) polyacrylamide gel (SDS-PAGE) to evaluate the expression of the protein and then transferred onto 0.22 *μ*m polyvinylidene fluoride (PVDF) membrane (Millipore, USA). After blocking with 5% nonfat milk, the membrane incubated with primary antibodies overnight at 4°C as follows: rabbit anti-NF-*κ*B p65 (1 : 1000), p-NF-*κ*B p65 (1 : 1000), p-I*κ*B-*α* (1 : 1000), I*κ*B-*α* (1 : 1000), p-Akt (1 : 1000), *β*-actin (1 : 3000), and PCNA (1 : 1000). After washing for 3 × 5 min in PBST (contain 1‰ tween20), goat anti-rabbit IgG (H&L) secondary antibody conjugated (1 : 5000) was incubated for 1 h at RT. Then, results were detected using an Odyssey Infrared Imaging System CLX-0796 (LI-COR, Lincoln, NE, USA), and semiquantitative analysis was then performed using Image J software.

### 2.7. Enzyme-Linked Immunosorbent Assay (ELISA)

The HUVECs were treated with SDG or DMSO (1%) for 1 h and then treated with LPS or PBS for 24 h; the supernatant of the cells was collected. The concentrations of IL-1*β*, TNF-*α*, and IL-6 in supernatants were measured with specific ELISA kit (Cusabio Biotech, Wuhan, China) according to the manufacturer's protocol. The absorbance was measured at 540 nm in a microplate reader.

### 2.8. Statistical Analysis

Statistical analysis was performed using PRISM 8. The results were presented as the mean ± standard deviation (SD). The multiple comparison method following one-way analysis of variance was used for comparisons among different groups, and differences with *p* < 0.05 were considered statistically significant. At least three independent experiments were performed for each condition.

## 3. Results

### 3.1. SDG Inhibits HUVEC Viability and Reduces the Angiogenic Capacity of HUVECs

**Figure 1:**
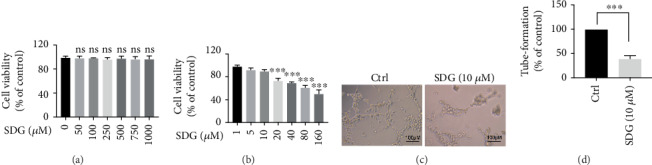
The effect of SDG on HUVEC viability reduces the angiogenic capacity of HUVECs. (a, b) HUVECs are treated with SDG in graded concentrations at lower doses (0, 50, 100, 250, 500, 750, or 1000 nM) and at higher doses (1, 5, 10, 20, 40, 80, and 160 *μ*M); then, cell viability was measured by CCK-8 assay. Compared to the 0 *μ*M or 1 *μ*M group, ∗∗∗ represents *p* < 0.0001. (c, d) SDG (10 *μ*M) reduces the angiogenic capacity of HUVECs. Error bars represent mean ± s.d. ∗∗∗ represents *p* < 0.001. All experiments were performed in triplicate. LPS: lipopolysaccharide; SDG: secoisolariciresinol diglucoside.

First, we investigated the effect of SDG on HUVEC viability using CCK-8 assay. HUVECs were treated with DMSO (1%) or SDG (lower doses: 0, 50, 100, 250, 500, 750, and 1000 nM; higher doses: 1, 5, 10, 20, 40, 80, and 160 *μ*M) for 24 h, and CCK-8 assays were conducted. The data showed that SDG did not exhibit toxicity at lower doses (*p* > 0.05; Figure 1(a)) and did not induce cell toxicity when its concentration is lower than 10 *μ*M (*p* > 0.05; Figure 1(b)). Therefore, 10 *μ*M SDG was used in the subsequent study.

Next, we tested the effect of SDG on HUVEC's angiogenic capacity using an endothelial cell tube-formation assay. The results showed that SDG significantly inhibited the network formation activity of HUVEC (*p* < 0.001; Figures 1(c) and 1(d)). Therefore, these findings indicate that SDG inhibits HUVEC viability and inhibits the angiogenic activity of HUVEC.

### 3.2. SDG Protects against LPS-Mediated HUVEC Injury and Apoptosis

**Figure 2:**
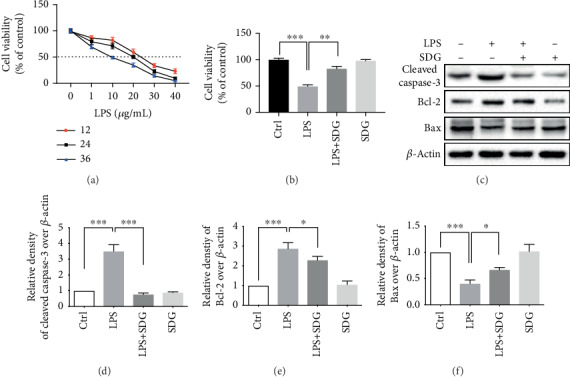
SDG mediated reduced LPS-mediated cell injury and apoptosis. (a) LPS (0, 1, 10, 20, 30, and 40 *μ*g/mL) was used to treat HUVECs for 12, 24, and 36 h. Cell viability was analyzed by CCK-8 assay. (b) Pretreated the cells with SDG (10 *μ*M) for 24 h, followed by LPS for 24 h. SDG increased the survival rate of cells in LPS-induced HUVECs. (c) Representative immunoblots of cleaved caspase-3, Bcl-2, and Bax proteins in different groups. (d–f) Semiquantitative analysis of the relative levels of cleaved caspase-3, Bcl-2, and Bax by a densitometric analysis. Error bars represent mean ± s.d. ∗, ∗∗, and ∗∗∗ represent *p* < 0.05, *p* < 0.01, and *p* < 0.001, respectively. All experiments were performed in triplicate. LPS: lipopolysaccharide; SDG: secoisolariciresinol diglucoside.

We evaluated LPS-induced HUVEC injury using the CCK-8 assay with LPS concentrations ranging from 0 to 40 *μ*g/mL for 12-36 h (Figure 2(a)). The data showed that the HUVEC viability reduced roughly 50% after 24 h treatment with LPS (20 *μ*g/mL) (Figure 2(a)). To explore the effect of SDG on HUVEC injury, we pretreated the cells with SDG (10 *μ*M) or DMSO (1%) for 24 h prior to adding LPS (20 *μ*g/mL) or PBS for 24 h. We found that SDG increased the survival rate of cells to 90% compared with LPS-induced HUVECs (*p* < 0.01; Figure 2(b)). This data suggests that SDG mediated significant cytoprotection LPS-mediated cell injury.

Then, the apoptosis induced by LPS in HUVECs was evaluated by western blot analysis (Figure 2(c)). The anti-cleaved caspase-3, anti-Bcl-2, and anti-Bax were used to assess apoptosis. The results revealed that SDG treatment could significantly downregulate the expression of cleaved caspase-3 (*p* < 0.001; Figure 2(d)) and Bcl-2 (*p* < 0.05; Figure 2(e)) and upregulate Bax (*p* < 0.05; Figure 2(f)) in LPS-mediated HUVECs. The above data suggest that SDG mediated reduced LPS-mediated cell apoptosis.

### 3.3. SDG Reduces NO Secretion in LPS-Treated HUVECs

**Figure 3:**
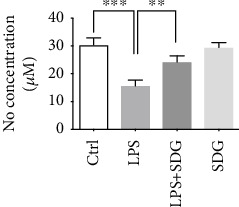
Effect of SDG on NO secretion in LPS-treated HUVECs. (a) SDG increased NO secretion in LPS-treated HUVECs. Error bars represent mean ± s.d. ∗∗ and ∗∗∗ represent *p* < 0.01 and *p* < 0.001, respectively. All experiments were performed in triplicate. LPS: lipopolysaccharide; SDG: secoisolariciresinol diglucoside.

Next, we investigated the effect of SDG on endothelial dysfunction by detecting HUVEC NO release which is a key mediator in LPS-stimulated HUVEC injury. The results showed that LPS decreased the production of NO compared with the control group (*p* < 0.001; Figure 3(a)). SDG significantly increased NO release compared with the control group (*p* < 0.01; Figure 3(a)). However, SDG treatment alone did not influence NO release (*p* > 0.05; Figure 3(a)). These results indicate that SDG exert protective effects against endothelial dysfunction by increasing NO secretion in LPS-treated HUVECs.

### 3.4. SDG Alleviated LPS-Induced Inflammation Response in HUVECs

**Figure 4:**
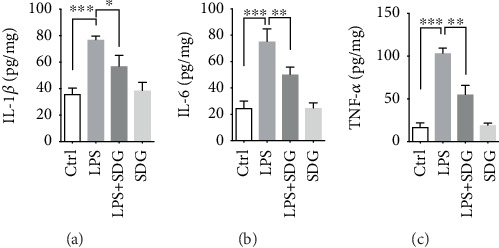
SDG alleviates LPS-induced inflammation response in HUVECs. (a–c) Semiquantitative analysis of the relative levels of IL-1*β*, IL-6, and TNF-*α* using ELISA. Error bars represent mean ± s.d. ∗, ∗∗, and ∗∗∗ represent *p* < 0.05, *p* < 0.01, and *p* < 0.001, respectively. All experiments were performed in triplicate. LPS: lipopolysaccharide; SDG: secoisolariciresinol diglucoside.

Studies have shown that SDG can regulate the immune response and reduce the inflammatory response to alleviate inflammatory bowel disease, but whether it can relieve endothelial damage by regulating the inflammatory response needs to be studied. Therefore, we examined the effect of SDG on LPS-induced inflammation response in HUVECs using ELISA assay (Figures 4(a)–4(c)). The results showed that LPS-induced significantly promoted the secretion of proinflammatory cytokines IL-1*β* (*p* < 0.001; Figure 4(a)), IL-6 (*p* < 0.001; Figure 4(b)), and TNF-*α* (*p* < 0.001; Figure 4(c)). SDG treatment significantly inhibited the expression of these cytokines due to LPS stimulation (*p* < 0.05; Figures 4(a)–4(c)). However, SDG treatment alone did not influence the expression of these cytokines (*p* > 0.05; Figures 4(a)–4(c)). These data suggest that SDG alleviates LPS-induced inflammation response in HUVECs.

### 3.5. SDG Inhibits the I*κ*B/NF-*κ*B Pathway in HUVECs

**Figure 5:**
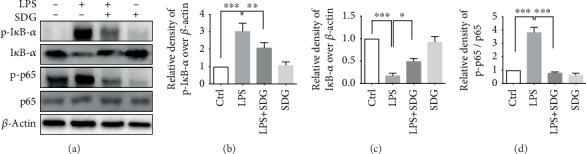
Effect of SDG on the I*κ*B/NF-*κ*B pathway in LPS-induced HUVECs. (a) Representative immunoblots of p-I*κ*B-*α*, I*κ*B-*α*, NF-*κ*B p65, and p-NF-*κ*B p65 proteins in different groups. (b–d) Semiquantitative analysis of the relative levels of p-I*κ*B-*α*, I*κ*B-*α*, and p-NF-*κ*B p65/p65 by a densitometric analysis. Error bars represent mean ± s.d. ∗, ∗∗, and ∗∗∗ represent *p* < 0.05, *p* < 0.01, and *p* < 0.001, respectively. All experiments were performed in triplicate. LPS: lipopolysaccharide; SDG: secoisolariciresinol diglucoside.

Our results showed that the levels of proinflammatory cytokines were significantly increased in LPS-induced inflammation response and inhibited by SDG in HUVECs. As we all know, these proinflammatory cytokines are key inflammatory cytokines of NF-*κ*B pathway-dependent cytokines. Therefore, we investigated whether SDG can inhibit the NF-*κ*B pathway. The protein expression of p-I*κ*B-*α*, I*κ*B-*α*, NF-*κ*B p65, and p-NF-*κ*B p65 was detected by western blot analysis. Meanwhile, the protein levels of p-I*κ*B-*α* and p-NF-*κ*B p65 in LPS-induced HUVECs were significantly increased (*p* < 0.001). However, such changes were reversed by SDG treatment (*p* < 0.01; Figures 5(a)–5(d)). However, SDG treatment alone did not influence the mRNA and protein expression of those proteins (*p* > 0.05; Figures 5(a)–5(d)). All these data indicated that SDG inhibited the I*κ*B/NF-*κ*B pathway in LPS-induced HUVECs.

### 3.6. SDG Inhibits the I*κ*B/NF-*κ*B Pathway by Downregulating Akt Expression in HUVECs

**Figure 6:**
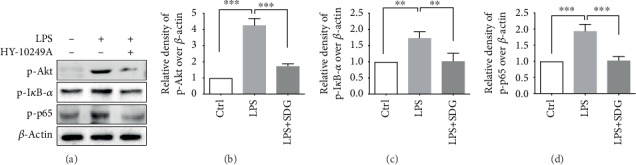
Effects of SDG on the protein expression of Akt in LPS-induced HUVECs. (a) Representative immunoblots of p-Akt, p-I*κ*B-*α*, and p-NF-*κ*B p65 in different groups. (b–d) Semiquantitative analysis of (a) by densitometric analysis. Error bars represent mean ± s.d. ∗∗ and ∗∗∗ represent *p* < 0.01 and *p* < 0.001, respectively. All experiments were performed in triplicate. LPS: lipopolysaccharide; SDG: secoisolariciresinol diglucoside.

We next investigated how SDG inhibits the I*κ*B/NF-*κ*B pathway in HUVECs. Phosphorylated Akt promotes the phosphorylation of I*κ*B. However, the mechanism by which Akt regulates NF-*κ*B expression in endothelial injury has not been studied. First, we inhibited the expression of Akt with HY-10249A (an inhibitor of Akt). Our results showed that p-Akt was significantly increased in LPS-induced HUVECs (*p* < 0.001) and decreased by SDG treatment (*p* < 0.001; Figures 6(a) and 6(b)). After treatment with HY-10249A, the expression of p-I*κ*B-*α* and p-NF-*κ*B p65 was reduced (*p* < 0.01; Figures 6(c) and 6(d)). The results suggested that Akt reduced the activation of I*κ*B-*α* and NF-*κ*B (*p* < 0.01; Figures 6(c) and 6(d)). All these data indicate that SDG inhibited the I*κ*B/NF-*κ*B pathway by downregulating Akt expression.

## 4. Discussion

In this study, we revealed that SDG inhibited LPS-mediated HUVEC injury and apoptosis. SDG could also reduce NO release and proinflammatory response in LPS-treated HUVECs. Meanwhile, SDG inhibited the NF-*κ*B pathway and downregulated Akt expression induced by LPS in HUVECs. Hence, these findings indicate that SDG exerts its cytoprotective effects in the context of LPS-treated HUVECs via regulation of the Akt/I*κ*B/NF-*κ*B pathway.

Endothelial injury is closely related to the progress of cardiovascular disease which is mainly caused by AS [17]. The endothelium is located in the innermost layer of the vasculature and directly interacting with various biochemical signals to maintain barrier function. It is involved in cell proliferation, senescence, cell death, and synthesizing a variety of functioning signaling molecules [18]. Dysregulated inflammatory response caused by infection or tissue damage is involved in the pathogenesis of many cardiovascular diseases and causes endothelial cell damage [19]. Repeated inflammatory stimuli lead to impaired vascular tone and increased permeability and vascular imbalance in endothelial cells, collectively contributing to the development of vascular disease [20]. Therefore, the pathophysiological stress response of endothelial cells caused by inflammation is closely related to cardiovascular disease. HUVEC is an endothelial cell which is a good model cell line for studying cardiovascular diseases especially AS. In our study, we founded that SDG has an effect on endothelial cell proliferation and angiogenesis, indicating that SDG may have some significance in stabilizing atheromatous plaques [21]. A study has confirmed that SDG prevents the oxidative stress-induced apoptosis by regulating the JAK2/STAT3 signaling pathway in myocardial cells [22] and abrogates the observed increases in ROS and apoptosis in cardiac iron overload condition [23]. Similarly, we found that SDG also inhibited LPS-mediated HUVEC injury and apoptosis. This result is also consistent with the result that SDG reduces space radiation-induced damage in lung endothelial cell injury [24].

NO is a regulator with important biological properties in endothelial cells [25]. It can regulate the tension of blood vessels and the local cell growth, as well as protect blood vessels from harmful molecular mediators, and maintain the dynamic balance of blood vessels and the normal endothelial function [26]. We found that SDG increased NO release in LPS-treated HUVECs, suggesting that SDG is involved in the balance of endothelial function by regulating NO secretion. Endothelial damage causes NO impaired synthesis or excessive oxidative degradation leading to endothelial dysfunction. Endothelial dysfunction triggers or exacerbates endothelial damage, which in turn leads to AS [27]. SDG protects cells by increasing NO secretion and may prevent endothelial damage. Moreover, NO metabolites can interact with endothelial damage, inflammation, and vascular disease [27, 28]. We also found that SDG inhibits the expression of proinflammatory cytokines, which is consistent with our previous and other findings [11, 29]. Studies have shown that immune inflammation is a key factor in the development of cardiovascular disease. Inflammation is a trigger for the early atherosclerosis process, and high expression of proinflammatory cytokines is associated with cardiovascular disease. IL-1*β* promotes inflammatory response and angiogenesis [30]. However, anti-inflammatory therapy targeting IL-1*β* clearly reduced the incidence of recurrent cardiovascular events by interfering with the innate immune pathway [31]. Prolonged TNF-*α* production is associated with endothelial dysfunction [32, 33]. Genetically determined reduced IL-6 signaling lowers the risk of multiple cardiovascular disease [34]. Therefore, SDG reduces IL-1*β*, TNF-*α*, and IL-6 expression, which indirectly reduce the damage of endothelial cells and the risk of cardiovascular disease.

NF-*κ*B, a central mediator of the inflammatory process, participates in innate and adaptive immune responses [35]. It is increasingly recognized as a crucial player in regulating inflammation and atherosclerosis [36]. Various tissue injuries or inflammatory stresses activate NF-*κ*B to trigger the NF-*κ*B signaling pathway, leading to damage to the vascular endothelium until the lesion develops to plaque formation and rupture [37, 38]. In our study, SDG inhibited the NF-*κ*B pathway in LPS-induced HUVECs, indicating that SDG affects atherosclerotic plaques may be through regulating the NF-*κ*B pathway. Importantly, we also found that SDG relieves LPS-mediated HUVEC injury partly dependent on the disruption of Akt activation. Silencing Akt reduces the level of the NF-*κ*B pathway. The Akt activates its downstream targets participating in cardiovascular processes by affecting cell survival, proliferation, angiogenesis, etc. [39]. This means that SDG's effect on LPS-mediated HUVECs mainly regulates the Akt/I*κ*B/NF-*κ*B pathway. These results are similar to this report that SDG is involved in the IGF/insulin signaling pathway and downregulated AKT expression [40]. This provides new insights studying SDG or the NF-*κ*B pathway in AS.

In our study, the results showed that SDG exerts its cytoprotective effects in the context of LPS-treated HUVECs via regulation of the Akt/I*κ*B/NF-*κ*B pathway, offering a new insight into our understanding of the molecular mechanism of AS. Therefore, SDG may be a promising therapeutic agent for the treatment of AS. However, further experimental research and rigorous clinical investigation should be conducted via human studies.

## Data Availability

All datasets generated for this study are included in the manuscript.

## Abbreviations

AS: Atherosclerosis
SDG: Secoisolariciresinol diglucoside
LPS: Lipopolysaccharide
HUVECs: Human Umbilical Vein Endothelial Cells
NO: Nitric oxide
IL-1*β*: Interleukin-1*β*
IL-6: Interleukin-6
TNF-*α*: Tumor necrosis factor-*α*
NF-*κ*B: Nuclear factor kappa-B
Akt: Protein kinase B
DMSO: Dimethyl Sulfoxide
h: Hour
ELISA: Enzyme-linked immunosorbent assay
RT: Room temperature.
